# The Complete Mitochondrial Genome and Phylogenetic Analysis of *Rhagastis binoculata* (Matsumura, 1909) (Lepidoptera: Sphingidae)

**DOI:** 10.3390/genes15091171

**Published:** 2024-09-06

**Authors:** Yu-Yun Kuo, Ju-Chun Chang, Yi-Hsuan Li, Yu-Feng Huang, Tzong-Yuan Wu, Yu-Shin Nai

**Affiliations:** 1 Department of Entomology, National Chung Hsing University, Taichung City 40227, Taiwan; yunhallo113gif@gmail.com (Y.-Y.K.); c1887g@email.nchu.edu.tw (J.-C.C.); lisa19980923@gmail.com (Y.-H.L.); yfhuang@csie.ntu.edu.tw (Y.-F.H.); 2 Doctoral Program in Microbial Genomics, National Chung Hsing University and Academia Sinica, Taichung City 402202, Taiwan; 3 Department of Bioscience Technology, College of Science, Chung Yuan Christian University, Zhongli District, Taoyuan City 32023, Taiwan

**Keywords:** mitogenome, Sphingidae, Macroglossinae, *Rhagastis binoculata*

## Abstract

The mitochondrial genome (mitogenome) *Rhagastis binoculata* (Matsumura, 1909), an endemic moth species in Taiwan, was sequenced and analyzed. The complete circular mitogenome of *R. binoculata* is 15,303 bp and contains 13 protein-coding genes, 22 transfer RNA genes, 2 ribosomal RNA genes, and an AT-rich control region. The mitogenome has an overall nucleotide composition of 41.2% A, 11.9% C, 7.5% G, and 39.4% T, with an AT content of 80.6%. Of the protein-coding genes (PCGs), 12 start with ATG, ATT, and ATC, and *COX1* starts with a “CGA” codon. All of the stop codons are “TAA, TAG, or T”. Our phylogenetic analysis of 21 species of Sphingidae insects suggests that *R. binoculata* is clustered with *Rhagastis mongoliana*, which belongs to the subfamily Macroglossinae.

## 1. Introduction

Lepidoptera is a large insect order with more than 160,000 species [1], including butterflies and moths. Many Lepidoptera species serve as important models in ecology and evolutionary biology [2]. In terms of lepidopteran insects, the mitochondrial genomes (mitogenomes) are characterized by a control region that is rich in adenine and thymine (A + T) and typically comprises 37 genes, including 13 conserved protein-coding genes (PCGs), 22 tRNAs, 2 rRNAs, and a non-coding region [3,4,5]. The size of the mitogenomes also varies from 15,000 to over 16,000 bp [3,4]. This variation in the mitogenomes is primarily due to differences in the lengths of non-coding regions, especially the control region [6]. Based on these characteristics, the mitogenomes of species in the order Lepidoptera are extensively used for research in population genetics, phylogeography, phylogenetics, and molecular taxonomy [7,8,9]. The mitogenome, in particular, is an excellent resource for phylogenetic analysis owing to its uncomplicated structure, maternal mode of inheritance, limited recombination, and significant evolutionary conservation [3,4].

The family Sphingidae, commonly known as hawk moths or sphinx moths, comprises a significant number of genera and species. There are approximately 200 genera and around 1350 species within this family [10], which is distributed globally, except in extreme environments like Antarctica and certain deserts [11]. Sphingidae moths play a crucial role in both ecological and economic contexts. Ecologically, they are important pollinators and serve as indicators of environmental health.

*Rhagastis binoculata* (Matsumura, 1909) is one species belonging to the family Sphingidae. It is an endemic moth species in Taiwan. According to recorded data, the *R. binoculata* species is distributed throughout low (ca. 161 m) to medium (ca. 2350 m) altitudes of mountain areas in Taiwan [12]. The males are easily drawn to light, whereas females are less frequently captured using light traps [13]. The wing length of *R. binoculata* ranges from 54 to 70 mm. *R. binoculata* adult males can be identified by a pinkish-gray oval patch that spans the postmedian lines and a more prominent black spot on the upper side of the forewing [13]. The underside of both the forewing and hindwing is generally orange in color, with patterns that are more diffused compared to those seen in *R. dichroae*, yet still discernible [13]. In contrast, the female shares similar traits with the male but features broader wings and a slightly lighter ground color. Additionally, the grayish zigzag medial line on the underside of both the forewing and hindwing is more noticeable in females [13]. The male genitalia of *R. binoculata* bear resemblance to those of *R. everetti*, though with some distinctive differences. The uncus and gnathos are comparatively shorter and thicker [13]. The valva is rounded, with the basal part narrower than the terminal part. The sacculus is very short, slightly curved, and ends in a blunt apex [13]. These anatomical details are essential for precise species identification and understanding the phylogenetic relationships within the genus *Rhagastis*.

The major host plants of *R. binoculata* larvae are the Chinese hydrangea (*Hydrangea chinensis*) and narrow-petaled hydrangea (*H. angustipetala*) [14]. Therefore, there is no record of agricultural losses in this species. The morphology of the *R. binoculata* larva consists of a green body color with distinctive white markings resembling clouds on its lateral sides. Positioned atop the hump behind the head are a pair of eyespots. Furthermore, a prominent central blue line runs along the dorsal part of the larval body. In this study, *R. binoculata* larvae displaying nucleopolyhedrosis were sampled from the Chinese hydrangea at Tianshang Mountain Trail, New Taipei City, Taiwan (24°57′26.0″ N 121°26′39.4″ E), at low altitude (ca. 400 m).

Nucleopolyhedrosis is a symptom caused by nucleopolyhedrovirus (NPV) infection. The NPV belongs to the genus *Alphabaculovirus* in the family Baculoviridae [15]. Based on our knowledge, baculoviruses are insect-specific pathogens, and over 600 insect host species have been reported for baculoviral infection [16]. Furthermore, since the 1980s, recombinant baculoviruses have been established as baculovirus expression vector systems (BEVSs) and widely applied to foreign protein expression in insects [16]. To date, the most commonly used commercial BEVSs are based on the Autographa californica multiple nucleopolyhedrovirus (AcMNPV) systems; however, the large-scale production of recombinant protein is still under development [17]. Baculoviruses usually exhibit a narrow host range [18]. In terms of host specificity, the identification of the insect species is merely important for the discovery of a new NPV infection in the field. Moreover, due to the large body size of *R. binoculata* larvae, it is considered to have the potential to be developed as a BEVS with highly foreign protein productivity. To explore this potential and to further understand the host of this NPV, the mitogenome of an *R. binoculata* larva was decoded with next-generation sequencing (NGS). These data could provide more information on the Macroglossinae subfamily and contribute to the study of the insect virus’s host range.

## 2. Materials and Methods

### 2.1. Sample Information, Collections, and DNA Extraction

In this study, a diseased *R. binoculata* larva, which showed nucleopolyhedrosis, was collected from the *H. chinensis* at Tianshang Mountain Trail, New Taipei City, Taiwan (24°57′26.0″ N 121°26′39.4″ E), at an altitude of ca. 400 m. The diseased-larva sample was deposited at the Insect Pathology and Genomics Laboratory (IPL) at the Department of Entomology, National Chung Hsing University, under voucher number NCHU_IPL_41. Total genomic DNA was extracted from the diseased *R. binoculata* larva using a genomic DNA extraction Kit (Geneaid Biotech Ltd., New Taipei City, Taiwan) following the user manual.

### 2.2. Genomic Sequencing and Data Analysis

**Figure 1:**
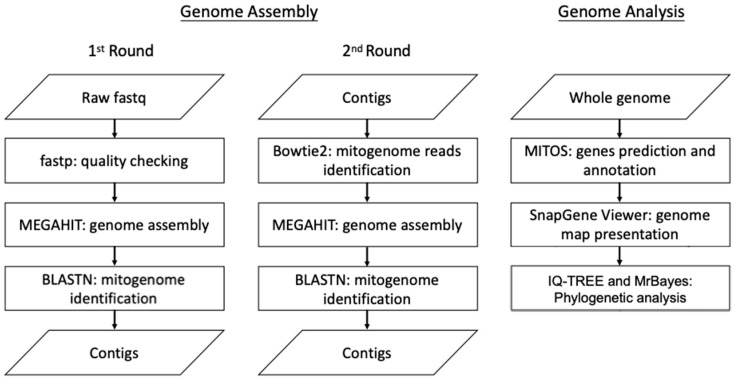
The workflow of genome assembly and analysis.

DNA sequencing was performed with an Illumina NovaSeq 6000 sequencer (Illumina, San Diego, CA, USA) with paired-end (PE) technology for 2 × 150 bp. The total PE reads were conducted for sequencing adapter identification and then trimmed with cutadapt [19]. Ambiguous bases and bases with lower quality values were removed with PRINseq [20] from either the 5′- or 3′ end. The quality check of the trimmed reads was performed using fastp [21]. These trimmed reads were then subjected to genome assembly and analysis. This workflow is summarized in Figure 1. The genome assembly was performed with a MEGAHIT de novo assembler with quality PE reads, as in a previous study [22]. Briefly, the quality reads were subjected to a 1st round of genome assembly by the MEGAHIT de novo assembler. The assembled contigs were then checked with NCBI BLASTn to identify the contigs related to the mitogenome sequences. All the quality reads were mapped to the 1st assembled mitogenome-related contigs, and the mapped reads were subjected to a 2nd round of genome assembly with the MEGAHIT de novo assembler to obtain the final genome draft. In total, four contigs were obtained, and their orientation was determined by aligning them against the reference mitogenome of *Clanis bilineata* (GenBank: MK804156.1). The draft mitogenome was then subjected to gap filling using PCR with four primer sets (Supplementary Table S1) to complete the circular mitogenome. The mitogenome of *R. binoculata* was annotated using MITOS WebServer (http://mitos.bioinf.uni-leipzig.de/index.py, accessed on 23 March 2023) [23], and the start and stop codons of the protein-coding genes (PCGs) were checked manually, in accordance with the study of Cameron et al., 2014 [24]. The genome map was presented by SnapGene Viewer (www.snapgene.com).

### 2.3. Species-Level Confirmation of Mitogenome

**Figure 2:**
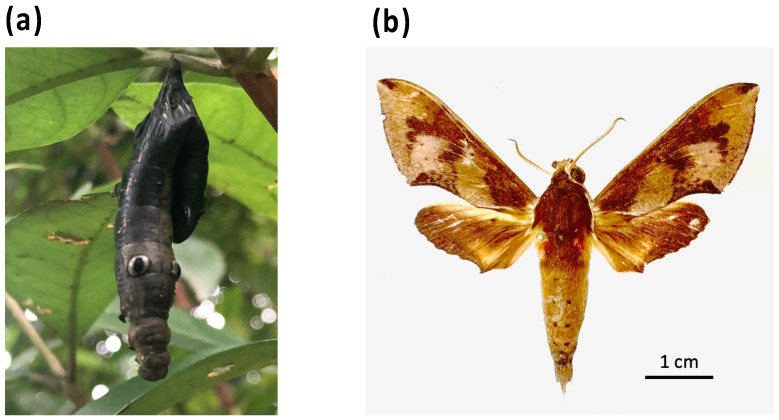
The larva and adult of *R. binoculata* (Matsumura, 1909). (**a**) The diseased larva (NCHU_IPL_41) with the symptom of nucleopolyhedrosis. (**b**) The adult specimen of *R. binoculata* (Matsumura, 1909) (NCHU_IPL_41-14) from the National Museum of Natural Science (NMNS).

To confirm that the mitogenome from the diseased *R. binoculata* larva (Figure 2a) was identical to that of the *R. binoculata* adult, an adult *R. binoculata* specimen was obtained from the National Museum of Natural Science (NMNS) under voucher number NCHU_IPL_41-14 (Figure 2b). DNA samples were extracted from both specimens and subjected to PCR amplification with a *COX1* primer set (Supplementary Table S1). The PCR products were then sequenced using Sanger sequencing, and the sequences were aligned with MAFFT (v7.551) (https://mafft.cbrc.jp/alignment/server/, accessed on 27 August 2024) to confirm the species of the diseased larva.

### 2.4. Phylogenetic Analyses

To better understand the classification of *R. binoculata*, a phylogenetic analysis of 21 moth species in Sphingidae was performed based on whole mitogenomes (Supplementary Table S2) [5,25,26,27,28,29]. To select the species of Sphingidae, the *COX1* gene was used to search the closely related species with NCBI BlastN [30], and species with a similarity between 90% and 100% were selected. The mitogenome of *C. bilineata* was also included in the phylogenetic analysis because the NPV found in *R. binoculata* is the most closely related to the NPV from *C. bilineata*. The control regions were trimmed before alignment using MUSCLE (v5.1) [31]. After alignment, the tree model was evaluated with ModelFinder [32,33], and then, the phylogenetic tree was constructed using IQ-TREE [34] with the model GTR + F + I + G4 and MrBayes 3.2.6 [35,36,37] with the model GTR + I + γ.

## 3. Results

### 3.1. Sequencing Summary, Mitogenome Assembly, and Annotation

**Figure 3:**
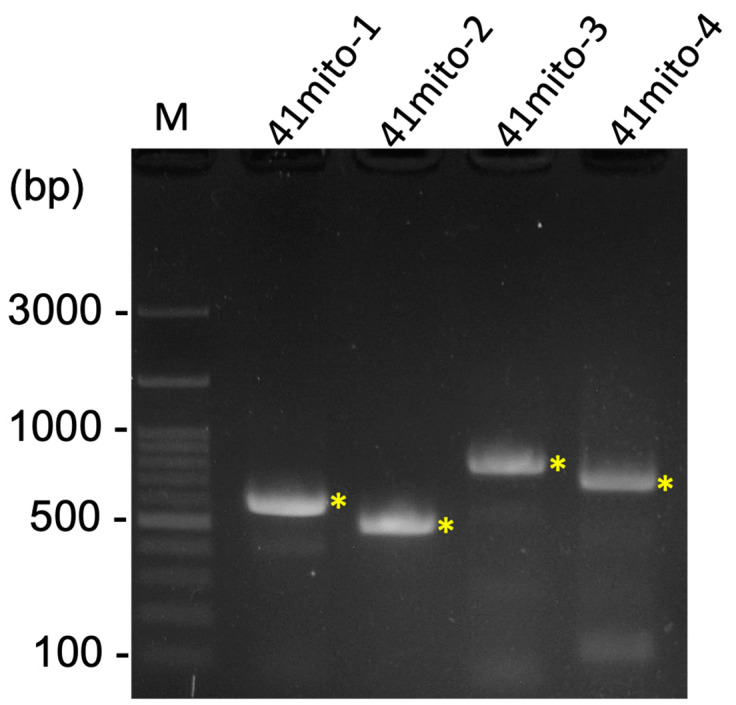
The result of four PCRs for the gap closing of the *R. binoculata* mitogenome. M = 100 bp DNA marker; bp = base pair; “*” indicates the target amplified PCR bands.

**Figure 4:**
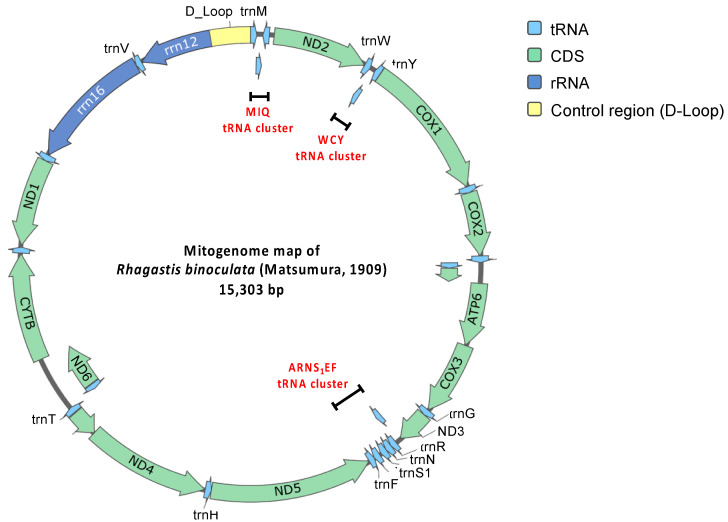
Circular mitogenome map of *R. binoculata* (Matsumura, 1909).

**Figure 5:**
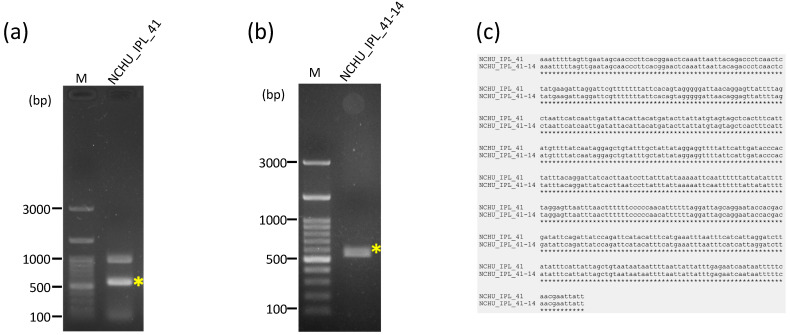
The confirmation of the identity of the diseased larva (NCHU_IPL_41) and adult *R. binoculata* specimen (NCHU_IPL_41-14) using the *COXI* gene PCR. (**a**,**b**), Sanger sequencing. (**c**). M = 100 bp DNA marker; bp = base pair; “*” indicates the target amplified PCR bands. The alignment of the sequences was performed by MAFFT (v7.551) https://mafft.cbrc.jp/alignment/server/, accessed on 27 August 2024.

A total of 62,429,098 clean raw reads were obtained through NGS, and the raw sequencing data were deposited in the NCBI database with the Sequence Read Archive (SRA) accession number PRJNA1045710. Of these reads, 59,799 reads belonged to the mitogenome of *R. binoculata*. After genomic assembly, four contigs were obtained, and the mitogenome was then concatenated and completed using PCR (Figure 3). The final completed mitogenome of *R. binoculata* was 15,303 bp in length with a 100% coverage and 107-fold mean depth (Supplementary Figure S1). The nucleotide composition was 41.2% A, 11.9% C, 7.5% G, and 39.4% T, with an A+T bias of 80.6% (Figure 4). In terms of the species-level confirmation, the *COX1* sequence of the diseased larva and the adult specimen of *R. binoculata* showed a 100% similarity in identity, indicating that these samples were the same species (Figure 5).

### 3.2. Protein-Coding Genes

The mitogenome consists of 13 protein-coding genes, 22 transfer RNA (tRNA) genes, 2 ribosomal RNA (rRNA) genes, and a major non-coding AT-rich control region (Table 1; Figure 4). Based on the comparison of 13 protein-coding genes, several start and stop codons of *R. binoculata* are different to those of *R. mongoliana*. For *ND2* (NADH dehydrogenase 2), the start codon (ATC) in the mitogenome of *R. binoculata* is different from that of *R. mongoliana*, which is ATT (start codon). For *COX1*, *COX2*, and *ND5* (NADH dehydrogenase 5), the stop codons (T) are different from those of *R. mongoliana* (ATT, CTT, and TTT, respectively). For *ATP8* (ATP synthase of subunit 8) and *ND5*, the start codons are ATT in *R. binoculata*, while the start codons found in *R. mongoliana* are ATC. The stop codon (TCT) of *ND5* in *R. binoculata* is different from that of *R. mongoliana* (stop codon = TTT). Moreover, the stop codon of *ND1* in *R. binoculata* is also different from that of *R. mongoliana*, which is TAA. These differences in codons usage might imply translational efficacy or RNA stability in different insect species.

### 3.3. Transfer RNA and Ribosomal RNA Genes

The mitogenome of *R. binoculata* has twenty-two tRNA genes and two ribosomal RNA genes (Figure 4). Among the tRNA genes, there are three tRNA clusters—MIQ, WCY, and ARNS_1_EF—at the genome positions of 1–199 bp, 1268–1454 bp, and 5919–6331 bp, respectively (Figure 4).

### 3.4. Phylogenetic Analysis

**Figure 6:**
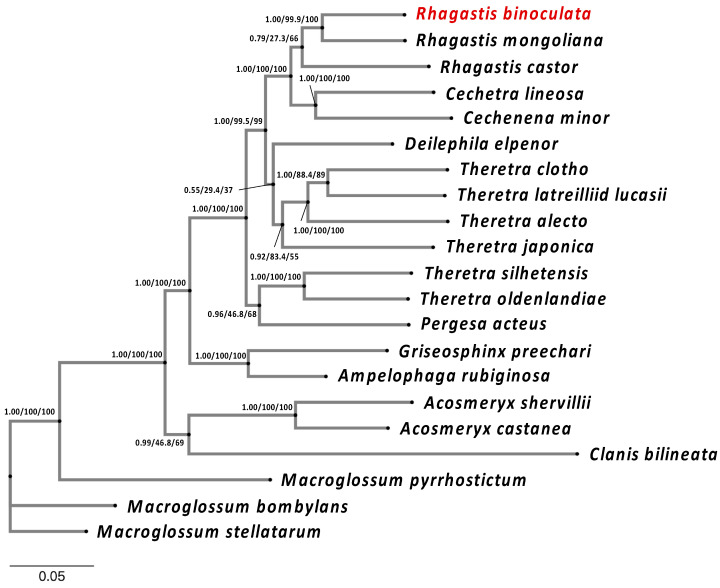
The phylogenetic analysis of *R. binoculata* (Matsumura, 1909) showing its relation to other close species. The phylogenetic analysis is based on whole mitogenomes, except for the control regions (A+T-rich) of 21 species in Sphingidae. The phylogenetic tree was constructed using both MrBayes and IQ-TREE, applying the Bayesian inference (BI) and maximum-likelihood (ML) methods. Branch support values are represented as Posterior Probability (PP) (for BI), UFBoot2 (for ML), and SH-Alrt (for ML) (PP/UFBoot2/SH-Alrt).

Both the MrBayes and IQ-TREE results indicate that *R. mongoliana* (GenBank: OL622048.1) is the closest species to *R. binoculata* (Figure 6) and that they both belong to the subfamily Macroglossinae. The phylogenetic trees from these two phylogenetic analyses had identical structures, which suggests that the results of the phylogenetic analysis of *R. binoculata* are reliable.

## 4. Discussion

After genomic sequencing, assembly, analysis, and PCR validation, the size of *R. binoculata*’s mitogenome is 15,303 bp. This mitogenome size is within the range of those of other Lepidopteran insects, which generally range from 15 to 16 kb [38]. The A+T content of insects varies, ranging from 64% (termites) to 86.7% (bees) [24]. Within the Lepidopteran species, the mitogenomes are A+T-rich (average% = 80.49% ± 0.95%), and also ranging from 76.01% (*Acraea polis*, Nymphalidae) to 83.13% (*Hesperia comma*, Hesperiidae) [39]. The A+T bias of *R. binoculata* mitogenome is 80.6%, aligning with the A+T richness typical of Lepidopteran species. All mitogenomes in Lepidoptera contain 37 genes and an AT-rich region, including 13 protein-coding genes (PCGs), 22 transfer RNA (tRNA) genes, and 2 ribosomal RNA (rRNA) genes. In *R. binoculata*, 23 genes are located on the forward strand, including 9 PCGs and 14 tRNA genes, while the remaining 14 genes are on the reverse strand. These characteristics have also been observed in previous research [38].

In typical Lepidopteran mitogenomes, the start codon of *COX1* is CGA, while the other 12 PCGs have ATN as their start codon [40]. However, there are some exceptions. The supposed start codon in *COX1* should be replaced by ATA or ATT in five primitive clades (superfamilies Adeloidea, Nepticuloidea and Hepialoidea) and some of Ditrysia (the superfamilies Tineoidea and Zygaenoidea). Additionally, the start codon “GTG” is partially found in the *COX2* and *ND1* genes of the superfamily Zygaenoidea [38] and is also present in other species’ mitogenomes [41]. In contrast to the diversity of start codons, there are only four types of stop codons: TAA, TAG, TA, and T [38]. TAA is the most common stop codon in PCGs. *COX1* and *COX2* use the incomplete codon T as stop codons, while the stop codon of *ND5* varies. These conserved mitogenomic features are common in invertebrates [42,43,44], and these non-standard stop codons (T and TA) can still be recognized by endonucleases during the transcription of polycistronic pre-mRNA, resulting in a functional stop codon through the polyadenylation of contiguous PCGs [45,46]. In this study of the start codons of PCGs in *R. binoculata*, the start codon of *COX1* is CGA, while the start codons of the other 12 codons are ATB (B=C or G or T). Among the stop codons, *COX1*, *COX2*, and *ND5* have incomplete stop codons (T).

There are 22 tRNA genes in the mitogenomes of *R. binoculata*. Fourteen of them are forward strand, while the remaining eight genes are reverse strand. Their lengths range from 64 to 71 bp. The shortest genes are *trnC*, *trnY*, *trnF*, and *trnS2*, and the longest gene is *trnK*. There are three tRNA clusters in *R. binoculata*, which are *trnM-trnI-trnQ*, *trnW-trnC-trnY*, and *trnA-trnR-trnN-trnS1-trnE-trnF*. In general, the *R. binoculata* mitogenome exhibits a structure congruent with the ancestral Pancrustacea model [47]. Given that the arrangement of genes in gene clusters is different in some species, this is one of the classification characteristics employed to distinguish between species. The gene order of *R. binoculata* is the same as that of other Sphingidae species and is typical of Lepidoptera [27,38]. Of the two rRNA genes, the large one is *rrn16* (1350 bp)*,* which is between *trnL1* and *trnV*, and the small one is *rrn12* (778 bp), which is located between *trnV* and the A+T-rich region. Both of the genes are reverse strand, which is identical to those in other sequenced hawkmoth genomes [27].

In the mitogenome of *R. binoculata*, there are a total of 8 overlapping regions involving 15 genes, and both the 3′ and 5′ ends of *trnT* overlap with two other genes. The largest region is 11 bp and is located between the 3′ end of *ND4L* and the 5′ end of *trnT*. The smallest overlaps, which are 1 bp, occur between the 3′ end of *trnT* and the 5′ end of *trnP*, the 3′ end of *trnK* and the 5′ end of *trnD*, and the 3′ end of *ND6* and the 5′ end of *CYTB*. Other overlapping regions are between the 3′ end of *trnI* and the 5′ end of *trnQ*, between the 3′ end of *trnW* and the 5′ end of *trnC*, between the 3′ end of *ATP8* and the 5′ end of *ATP6*, and between the 3′ end of *ND3* and the 5′ end of *trnA*, with a length of 3, 8, 7, and 2 bp, respectively. The overlap sequence 5′-ATGATAA-3′ between *ATP8* and *ATP6* is commonly found in all known Lepidopteran mitochondrial genomes [38,43,48,49].

The AT-rich region, which is also known as the control region, is located between *rrn12* and *trnM* and has an average length of 439 bp [50]. The length of the control region in the mitogenome of *R. binoculata* is 425 bp. This region is considered to be associated with the origin of replication and transcription [24,47]. This region is characterized by a high content of adenine and thymine, with the control region of *R. binoculata* having an A+T content of 92.48%. Discrepancies in the start and stop codons suggest the potential divergence between *R. binoculata* and *R. mongoliana*. Phylogenetic analysis indicates that *R. binoculata* and *R. mongoliana* are clustered together within the Macroglossinae subfamily, emphasizing their similarity in evolution. Although the phylogenetic trees analyzed using MrBayes and IQ-TREE have identical structures, indicating a certain level of reliability, some nodes have branch support values where ML is less than 50 and PP is less than 0.5. This suggests that the classification of these branches is not very stable. It is hoped that with the release of more genetic sequencing data, these issues can be resolved in the future.

In summary, this study provides crucial genetic insights into the endemic moth species *R. binoculata*, offering a foundation for future research on its ecological and molecular aspects. Our comparative genomics and phylogenetic analysis contribute valuable resources for broader studies on the evolutionary dynamics and biodiversity within the Sphingidae family. In the future, the practical application of *R. binoculata* in the BEVS will undergo further testing and evaluation.

## Figures and Tables

**Table 1:** The mitochondrial genes of the *Rhagastis binoculata* mitogenome.

Gene	Position		Strand	Length (bp)	Overlapping (bp)	Start Codon	Stop Codon	Anti-Codon
	Start	Stop						
*trnM*	1	68	F	68	-	-	-	AUG
*trnI*	69	133	F	65	3	-	-	AUC
*trnQ*	131	199	R	69	3	-	-	CAA
*ND2*	252	1265	F	1014	-	ATC	TAA	-
*trnW*	1268	1334	F	67	8	-	-	UGA
*trnC*	1327	1390	R	64	8	-	-	UGC
*trnY*	1391	1454	R	64	-	-	-	UAC
*COX1*	1458	2988	F	1531	-	CGA	T	-
*trnL2*	2989	3055	F	67	-	-	-	UUA
*COX2*	3056	3737	F	682	-	ATG	T	-
*trnK*	3738	3808	F	71	1	-	-	AAG
*trnD*	3808	3873	F	66	1	-	-	GAC
*ATP8*	3874	4035	F	162	7	ATT	TAA	-
*ATP6*	4029	4703	F	675	7	ATG	TAA	-
*COX3*	4707	5498	F	792	-	ATG	TAA	-
*trnG*	5501	5566	F	66	-	-	-	GGA
*ND3*	5567	5920	F	354	2	ATT	TAG	-
*trnA*	5919	5984	F	66	2	-	-	GCA
*trnR*	5985	6049	F	65	-	-	-	CGA
*trnN*	6050	6116	F	67	-	-	-	GUU
*trnS1*	6117	6183	F	67	-	-	-	AGC
*trnE*	6201	6267	F	67	-	-	-	GAA
*trnF*	6268	6331	R	64	-	-	-	UUC
*ND5*	6332	8069	R	1738	-	ATT	T	-
*trnH*	8070	8135	R	66	-	-	-	CAC
*ND4*	8141	9472	R	1332	-	ATG	TAA	-
*ND4L*	9474	9764	R	291	11	ATG	TAA	-
*trnT*	9774	9839	F	66	11,1	-	-	ACA
*trnP*	9839	9903	R	65	1	-	-	CCA
*ND6*	9912	10,442	F	531	1	ATG	TAA	-
*CYTB*	10,442	11,590	F	1149	1	ATG	TAA	-
*trnS2*	11,597	11,660	F	64	-	-	-	UCA
*ND1*	11,681	12,616	R	936	-	ATG	TAG	-
*trnL1*	12,618	12,684	R	67	-	-	-	CUA
*rrn16*	12,685	14,034	R	1350	-	-	-	-
*trnV*	14,036	14,100	R	65	-	-	-	GUA
*rrn12*	14,101	14,878	R	778	-	-	-	-
D_Loop	14,879	15,303	-	425	-	-	-	-

## Data Availability

The sequencing raw data were deposited in the NCBI (https://www.ncbi.nlm.nih.gov, accessed on 28 November 2023) database with the Sequence Read Archive (SRA) accession number PRJNA1045710. The mitogenome data are available in the NCBI database under the GenBank reference number OQ812083.1.

## Supplementary Materials

The following supporting information can be downloaded at https://www.mdpi.com/article/10.3390/genes15091171/s1: Supplementary Figure S1: Coverage plot of *R. binoculata* mitogenome; Supplementary Table S1: Primer sets in this study; Supplementary Table S2: The mitogenome sequence used in this study.
