# Supplementary figures and images for: The Complete Mitochondrial Genome and Phylogenetic Analysis of Rhagastis binoculata (Matsumura, 1909) (Lepidoptera: Sphingidae)
Source: Genes (Basel). 2024 Sep 6;15(9):1171. doi: 10.3390/genes15091171 (PMC11430935; doi:10.3390/genes15091171)

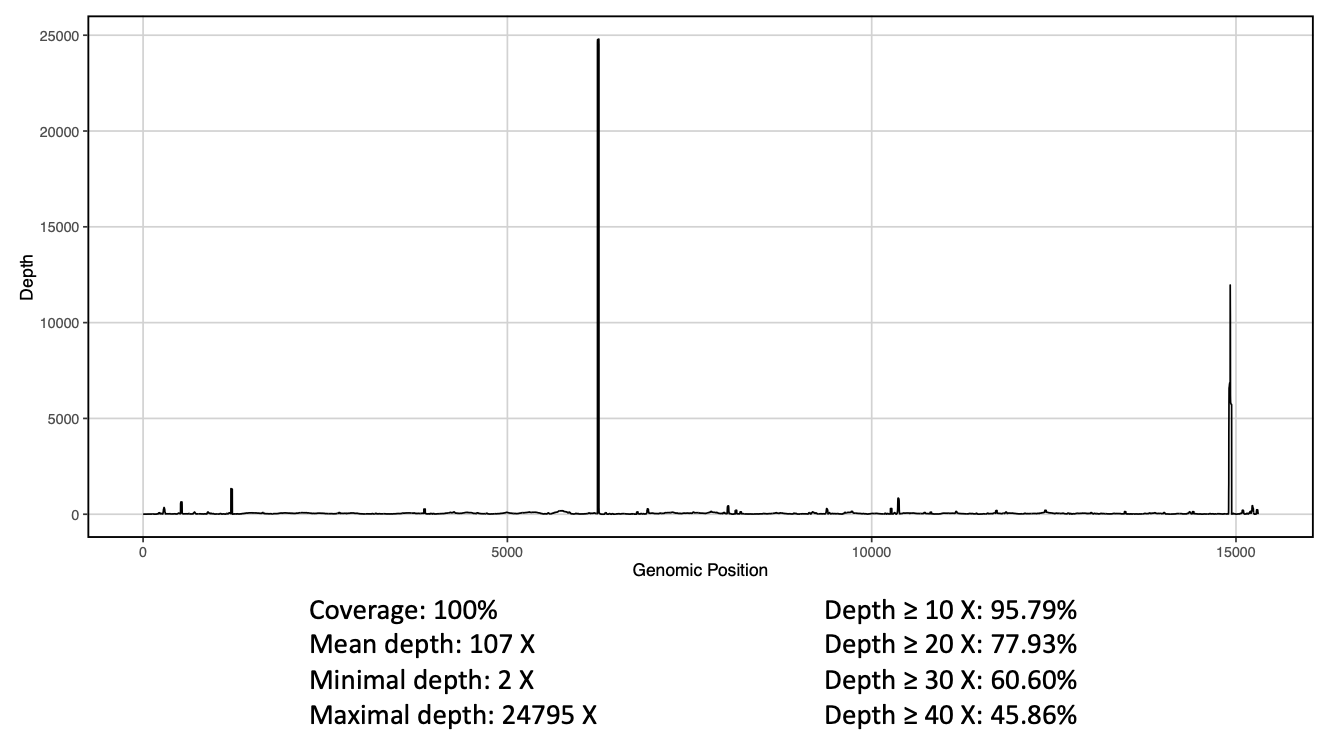

Supplement: Supplementary file 1 [file genes-15-01171-s001.zip › Supplementary_Figure_1.tiff]
